# Feeding practices and growth among young children during two seasons in rural Ethiopia

**DOI:** 10.1186/s40795-017-0158-y

**Published:** 2017-04-24

**Authors:** Mekitie Wondafrash, Lieven Huybregts, Carl Lachat, Kimberley P. Bouckaert, Patrick Kolsteren

**Affiliations:** 1 Department of Population and Family Health, College of Health Sciences, Jimma University, P.O. Box 343, Jimma, Ethiopia; 2 Department of Food Safety and Food Quality, Faculty of Bioscience Engineering, Ghent University, Coupure Links 653, 9000 Ghent, Belgium; 3 Poverty, Health and Nutrition Division, International Food Policy Research Institute, 2033 K Street NW, 20006 Washington DC, USA; 4 Unit of Nutrition and Child Health, Institute of Tropical Medicine, Nationalestraat 155, 2000 Antwerp, Belgium

**Keywords:** Ethiopia, Dietary diversity, Infant and child feeding index, Growth, Season

## Abstract

**Background:**

The use of indices of infant and young child feeding practices to predict growth has generated inconsistent results, possibly through age and seasonal confounding. The aim of this study was to evaluate the association of a dietary diversity score (DDS) and infant and child feeding index (ICFI) with growth among young children in a repeated cross-sectional and a follow-up study in two distinct seasons in rural southwest Ethiopia.

**Methods:**

We used a repeated cross-sectional design comparing child feeding practices to nutritional status in 6–12 month old children during harvest (HS; *n* = 320) and pre-harvest season (PHS; *n* = 312). In addition, 6–12 month old children from the HS were reassessed 6 months later during PHS. In addition to child anthropometry, child feeding practices were collected using 24-h and 7-day dietary recalls.

**Results:**

The mean (±SD) length-for-age z-score (LAZ) of the 6–12 month old children was −0.77 (±1.4) and −1.0 (±1.3) in HS and PHS, respectively, while the mean (±SD) of the follow-up children in PHS was −1.0 (±1.3). The median DDS (IQR) was 2.0 (1.0, 3.0.), 2.0 (2.0, 3.0) and 3.0 (2.0, 4.0) for the children in HS, PHS and the follow-up children in PHS, respectively. The DDS in HS was positively associated with LAZ at follow-up (β = 0.16; 95% CI: 0.01, 0.30; *P* = 0.03) after controlling for confounding factors. ICFI and DDS were not associated with mean LAZ, weight-for-height z-score and weight-for-age z-score within season. However, the odds of being stunted when having a DDS ≤ 2 was 2.3 times (95% CI: 1.10, 4.78; *P* = 0.03) higher compared to a DDS > 2 child in HS and 1.7 times (95% CI: 1.04, 2.71; *P* = 0.04) higher for the pooled sample of 6–12 months old children in HS and PHS.

**Conclusions:**

The DDS was found to be an indicator for child stunting during the Ethiopian harvest season. The DDS can be an appropriate tool to evaluate the association of child feeding practices with child growth irrespective of season. Inclusion of other dimensions in the construction of ICFI should be considered in future analysis as we found no association with growth.

**Electronic supplementary material:**

The online version of this article (doi:10.1186/s40795-017-0158-y) contains supplementary material, which is available to authorized users.

## Background

Child undernutrition is persistent in low income countries [1–3]. Ethiopia has one of the highest rates of childhood stunting despite a reduction since 2000 [4]. Among others, suboptimal child feeding practices are important underlying determinants of global poor child growth [5]. Of the children who are breastfed, only a third of children 0–6 and 6–23 months old are exclusively breastfed and receive complementary food respectively [4]. Moreover, only 4% of children 6–23 months old are fed as per the global recommendations.

The construction of summary child feeding indices to assess nutritional outcomes has gained momentum since the early 2000’s. The infant and child feeding index (ICFI) was developed based on breast feeding, bottle feeding, feeding frequency and dietary diversity in the previous 24 h and consumption of food groups in the previous 7 days. It has been suggested that infant and child feeding practices tend to cluster in which earlier good practices by a caregiver are more likely to continue later with more awareness on appropriate feeding behaviors [6]. Hence, data on child feeding practices collected over a short period of time were reported to indicate longer term health and nutritional outcomes. On the other hand, a previous review questioned the strength of several existing dietary quality indices in terms of their diagnostic capacity of various health outcomes [7]. Demographic and Health Survey (DHS) data have shown that dietary diversity alone was also associated with height -for-age z-score (HAZ) in 10 out of the 11 countries analyzed [8]. Nevertheless, the associations of infant and child feeding practices with HAZ were not consistent across geographical locations and age categories. The majority of studies showed associations for children aged 12 months and older and not for children in their first year of life. The arbitrary selection of components and cutoffs, as well as determining the contribution of components to the overall index have been highlighted as limitations to predict specific health outcomes or nutritional status [9].

Summary indices that include more information on feeding practices as a whole, in contrast to the diet quality itself, were reported to predict nutritional status [6]. Several evaluations have shown a positive association of such summary indices of child feeding practices with linear growth, i.e. height/length-for-age z-score (HAZ/LAZ) or stunting (HAZ/LAZ < −2) [10–14]. Conversely, the associations between child growth and individual components of the summary feeding index, i.e. breast feeding or bottle feeding alone, were found to be rather incoherent.

Adding to the complexity of associations between dietary quality or feeding practices indicators and child nutritional status are the profound seasonal effects in Sub-Saharan Africa on food availability and accessibility. To our knowledge, no study has examined the impact of seasonality on these associations. Previously, we found an association of DDS with mean micronutrient density adequacy (MMDA) and the association proved to be stable over two distinct seasons in Southwest Ethiopia [15]. Moreover, having a DDS ≤ 2 was associated with an inadequate intake of micronutrients. The aim of this study was to assess the association of ICFI and DDS with growth among infants and young children. As a first step, we analyzed the associations between ICFI and DDS with child growth using repeated cross-sectional data from two distinct seasons to assess the impact of seasonality on the strength of the association in children 6–12 months of age. Secondly, we assessed the association of ICFI and DDS with child growth longitudinally using follow-up data.

## Methods

### Study setting

The study was conducted in nine kebeles (smaller government unit) of the catchment area of Gilgel Gibe Field Research Center of Jimma University, Southwest Ethiopia. The Research Center was established in 2005 to serve as one of the Demographic Surveillance Systems in Ethiopia and field epidemiology attachment site of Jimma University. According to the census conducted in 2008, the total population within the area was 48,316 of which 49.4% were males with an average household size of 4.7 (SD = 2.2). One third of the households were urban [16]. The main source of income for the community is farming and raising livestock. Maize, sorghum and Teff (a species of *Eragrostis* native to Ethiopia) are commonly grown food crops in the area. Food consumption patterns of the population are expected to be different based on the differences in food availability in the two seasons, i.e. harvest (HS) and pre-harvest (PHS) season.

### Sampling and design

We combined two study designs. Firstly, a repeated cross-sectional survey was conducted using a census of all children aged 6–12 months within the study area during HS, from October to December 2009, and PHS, from June to August 2010 [17]. Children aged 6–12 months old and born in the area, who have a biological mother or female caregiver, presenting no serious illnesses which warrant immediate medical attention or referral (e.g. rapid breathing, nasal flaring, reported high fever, abnormal body movement, persistent diarrhea (≥2 weeks) or vomiting, etc.) or malformations affecting anthropometric measurement, and no intention of leaving the area in the coming 6 months were invited to participate. A total of 320 and 312 children were eligible in the HS and PHS, respectively.

**Fig. 1:**
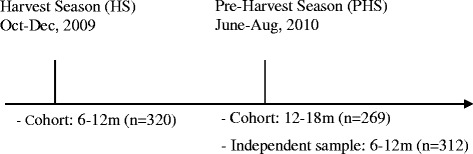
Study design and age groups of different samples

Secondly, we conducted a follow-up or cohort study of children who participated in the cross-sectional survey in HS. The follow-up was done 6 months later, i.e. in PHS from June to August 2010 (Fig. 1).

### Data collection

We used pre-tested interviewer-administered questionnaires to collect data on socio-economic status, demographics and infant and young child feeding practices (Additional file 1). Questions were adapted from the Ethiopian Demographic and Health Survey (EDHS) questionnaires [18] and the World Health Organization (WHO) guideline for the assessment of infant and young child feeding [19]. Data collectors with previous experience of data collection in the research center and able to fluently speak the local languages (Amharic and Afan Oromo) were recruited and received a 5-day intensive training from the principal investigators.

### Dietary diversity score

Dietary diversity was measured based on the method suggested by Dewey et al. [20]. The DDS was obtained by summing the number of food groups consumed in the previous 24 h by the infant. The following seven food groups were included: a) Grains, roots and tubers; b) legumes and nuts; c) dairy products; d) flesh foods; e) eggs; f) vitamin A-rich fruits and vegetables (>130 retinol equivalents/100 g); g) Other fruits and vegetables. Dietary diversity score was used in our analyses as it is or dichotomized. Previous analyses showed that a DDS ≤2 is associated with a low micronutrient intake [15, 20, 21] which warrants the investigation of its association with growth of infants and young children.

### Construction of the infant and child feeding index

The ICFI was calculated using core indicators as described previously [21, 22]. Dietary diversity was assessed using the method described above and age-specific scores were assigned (Table 1). As there was no information on energy density of complementary foods or breast milk intake, the scoring given to feeding frequency is based on the previous recommendation [23] which assumes low energy density and average breast milk intake. A score of +1 was attributed for those who met the lower end of the recommendation and +2 for those who exceeded the recommendation. Food group frequency was assessed using a questionnaire developed specifically for this purpose in which we asked the number of days a specific food group was consumed. Frequency was then scored 0 if not consumed during the previous week, +1 if consumed on 1–3 days and +2 if consumed for ≥4 days. These scores were summed up yielding a possible range of 0–14. Subsequently, new scores were assigned depending on age category as shown in Table 1. Each component of ICFI was defined and scored according to the current age-specific infant and young child feeding recommendations [24]. The ICFI was calculated by adding up all the age-specific scores. The index was cut into tertiles in which a score of 0–5 was considered low, 6–7 as medium and 8–9 as high, as described previously by Moursi et al. [13]. The index was also treated as a continuous variable in our analyses.

**Table 1:** Scoring system used to create the infant and child feeding index for children aged 6–12 months

Components	Age-specific scores	
	6–8 months	9–12 months
Breast feeding	Yes =2 No = 0	Yes =2 No = 0
Bottle feeding	Yes =0 No = 1	Yes =0 No = 1
Dietary diversity (24 h)	0–1 = 0 2 = 1 3 + = 2	0–2 = 0 3 = 1 4 + = 2
Food group frequency (previous 7 days)	0–2 = 0 3–4 = 1 5 + = 2	0–3 = 0 4–5 = 1 6 + = 2
Feeding frequency	0–1 = 0 2 = 1 3 + = 2	0–2 = 0 3 = 1 4 + = 2

### Anthropometry

Anthropometric measurements were standardized through repetitive exercises. Recumbent length was measured on an infant measuring board and recorded to the nearest 0.1 cm (SECA 210, Hamburg, Germany). Child’s weight was measured naked or with very light clothes using mother-child digital scales and recorded to the nearest 0.1 kg (SECA Uniscale, Hamburg, Germany). Age was recorded from birth certificates or immunization cards as much as possible. If reliable documents for age estimation were absent, local events calendars were used to help the mother or caregiver estimate the approximate age of the child. Most of the anthropometric data collectors in HS were also available in PHS.

### Household socio-economic status

We constructed an asset-based proxy to estimate the socio-economic status (SES) of the included households. Items included were ownership of cattle, house construction materials, land (homestead, land under cultivation, fallow land) and ownership of other household assets. For household assets, categories for each item were ‘owned’ or ‘not owned’ by the household. The first principal component (56% of the variance) from a principal components analysis was divided in tertiles and used as a proxy for SES [25].

### Morbidity

Morbidity was assessed qualitatively by asking the mother about the presence of diarrhea (defined as ≥3 episodes of loose stool per day), fever and cough (yes/no) in the previous 2 weeks of the survey.

### Data analysis

The anthropometric measures were converted into z-scores of weight-for-age (WAZ), length-for-age (LAZ) and weight-for-length (WLZ) based on the WHO 2006 Child Growth Standards [26]. Underweight, wasting and stunting were defined as WAZ, WLZ and LAZ < −2 z-scores from the median of the standard, respectively [27]. Descriptive analyses were conducted for SES and demographic data, morbidity, maternal schooling, household characteristics, nutritional status, dietary diversity and feeding practices of children aged 6–12 months for the repeated cross-sectional study. Demographic data, morbidity, nutritional status and dietary diversity were described separately for the follow-up children aged 12–18 months.

Means (±SD) were reported for continuous data which were normally distributed, otherwise the median (IQR) was used. Differences in means were statistically tested using a student t-test for normally distributed outcomes or Wilcoxon-Mann–Whitney test for skewed outcomes, while a *χ* ^2^-square test was used to compare categorical variables. The DDS was used as a standalone outcome and as a component of ICFI in the analyses. We used multiple linear regression models to analyze the association between DDS and ICFI with LAZ, WLZ and WAZ adjusted for SES and demographic data, morbidity status, maternal schooling of the 6–12 month old children in the repeated cross-sectional studies and follow-up children. Multiple logistic regressions were used to model DDS and ICFI with binary categories of LAZ, WLZ and WAZ (cut-off value: −2 SD). Bivariate analyses were performed between all predictor variables and anthropometric outcomes and then every variable with a modest association (*P* < 0.25) was a candidate to be included in the multivariate analyses. A full model assessing the relation between DDS, ICFI and components of ICFI was compiled after which non-significant variables were removed by a backward procedure using a likelihood ratio test (*P* < 0.05).

The internal consistency of the ICFI was checked by using both the Fisher exact test of association of ICFI (categorized) with its components being bottle feeding, food group frequency and feeding frequency, and by the Cronbach α coefficient.

As there was no effect of season on the association of DDS and ICFI and child growth or nutritional status, the data of the two cross-sectional studies were pooled and the same analysis was performed to increase power adjusting for season and the abovementioned confounding variables. As the interaction of DDS or ICFI with season was not significant (*P* >0.1), the interaction term was not included in the models. Moreover, a DDS cutoff of ≤2 was used to dichotomize DDS based on a prior sensitivity and specificity analysis [15] and its association with child anthropometry was tested.

ANCOVA was used to assess the longitudinal relationship of anthropometric indices with DDS and ICFI in the follow-up children [28]. For this purpose anthropometric indices at follow-up (PHS) were used as outcome variables and anthropometric indices at baseline (HS) as predictor variables. The DDS and ICFI at HS were also entered separately in the models as predictor variables. Data was analyzed with Stata 13.0 (StataCorp, College Station, TX, USA). Statistical significance was set at 5% and all tests were two-sided.

## Results

In the repeated cross-sectional surveys in HS and PHS, data was collected from 320 and 312 children aged 6–12 months respectively. In total, 24 children in HS and 21 children in PHS who had incomplete complementary food intake data and/or information pertaining to breast feeding (HS =21; PHS =15) or uncertain age (HS =3; PHS =6) were omitted from analysis. The mean LAZ, WAZ and WLZ of excluded observations did not differ statistically from the rest of the observations in both seasons.

Of the 296 children with complete data at HS, 27 (9%) children could not be traced after repeated visits at follow-up. Three observations from the follow-up children were excluded from analysis as they did not have complete dietary diversity data.

### Nutritional status

In the repeated cross-sectional surveys in HS and PHS, 6–12 month old children had a stunting prevalence of 18.6 and 21.1% respectively, and a wasting prevalence of 11.8 and 15.5% respectively. The mean LAZ was −0.77 ± 1.4 and −1.0 ± 1.3 for the children in HS and PHS respectively. At follow-up, 21% of 12–18 month old children were stunted and 20% were wasted, whereas the mean LAZ was −1.0 ± 1.3 (Table 2).

**Table 2:** Characteristics of 6–12 month old children in the repeated cross-sectional and follow-up study in HS and PHS

Variables	HS (*n* = 296)	PHS (*n* = 291)	*P*-value^*^	Follow- up at PHS (*n* = 266)
Gender of child				
Female	154 (52.0)	125 (43.0)	0.03	137 (51.5)
Age of the child (6–12)				-
6-8 months	152 (51.4)	71 (24.4)	<0.001	
9–12 months	144 (48.6)	220 (75.6)		
Age of the child (12–18)				
12–15 months				183 (68.8)
16–18 months				83 (31.2)
Age of the mother				-
< 20 years	60 (20.6)	32 (11.0)	˂0.01	
20–34 years	202 (69.4)	233 (80.1)		
≥ 35 years	29 (10.0)	26 (8.9)		
Socioeconomic status, *n* (%)				-
Low	79 (27.3)	115 (39.9)	0.01	
Medium	106 (36.7)	89 (30.9)		
High	104 (36.0)	84 (29.2)		
Under-five children in the HH, *n* (%)				-
0–1	79 (26.7)	66 (22.7)	0.26	
≥ 2	217 (73.3)	225 (77.3)		
Morbidity, *n* (%)				
Cough^a^	44 (14.9)	57 (19.6)	0.13	36 (13.5)
Fever^a^	57 (19.3)	67 (23.0)	0.27	53 (19.9)
Diarrhea^a^	49 (16.7)	70 (24.1)	0.03	67 (25.2)
Source of drinking water for HH, *n* (%)				-
Unsafe water source	252 (85.1)	229 (78.7)	0.04	
Human waste disposal by the HH, *n* (%)				
Improper disposal	117 (39.7)	97 (33.3)	0.11	
Maternal schooling, *n* (%)				-
No formal schooling	243 (82.1)	246 (84.5)	0.43	
LAZ, mean ± SD	−0.77 ± 1.4	−1.0 ± 1.3	0.02	−1.0 ± 1.3
Stunting, *n* (%)				
LAZ < −2	55 (18.6)	61 (21.1)	0.44	55 (20.7)
LAZ < −3	18 (6.1)	16 (5.5)	0.78	16 (6.0)
WLZ, mean ± SD	−0.63 ± 1.3	−0.75 ± 1.3	0.28	−0.81 ± 1.2
Wasting, *n* (%)				
WLZ < −2	35 (11.8)	45 (15.5)	0.19	51 (19.2)
WLZ < −3	12 (4.1)	12 (4.1)	0.96	11 (4.1)
WAZ, mean ± SD	−0.96 ± 1.2	−1.1 ± 1.4	0.08	−1.1 ± 1.1
Underweight, *n* (%)				
WAZ < −2	52 (17.6)	75 (25.8)	0.02	49 (18.4)
WAZ < −3	18 (6.1)	20 (6.9)		13 (4.9)
Current breast feeding				
Yes	296 (100)	291 (100)	-	253 (95.1)
DDS, median (IQR)	2.0 (1.0, 3.0)	2.0 (2.0, 3.0)	0.17	3.0 (2.0, 4.0)
ICFI, median (IQR)	5.0 (5.0, 7.0)	6.0 (5.0, 7.0)	0.09	-

*HS* harvest season, *PHS* pre-harvest season, *HH* household, *LAZ* length-for-age z-score, *WLZ* weight-for-length z-score, *WAZ* weight-for-age z-score, *DDS* Dietary diversity score, *ICFI* Infant and Child Feeding Index

^*^ *P*-values are calculated for the difference of the two independent samples at HS and PHS

^a^Diarrhea, cough, fever in the previous 2 weeks of the studies

### Child feeding practices

All children aged 6–12 months were partially breastfed at the time of the surveys. The median (IQR) recalled duration of exclusive breastfeeding was 4.0 (3.0, 4.0) and 4.0 (3.0, 6.0) months in HS and PHS respectively; while 19 and 11% of these children were bottle-fed during HS and PHS respectively. Eighty-eight and sixty seven percent (*P* < 0.05) of the children were introduced to complementary foods before 6 months of age in HS and PHS respectively. There was no significant difference in DDS (*P* = 0.17) and ICFI (*P* = 0.09) between the two seasons (*P* = 0.17) for children aged 6–12 months (Table 2).

The follow-up data showed that breastfeeding was continued well into the second year of life. Only 4.8% of the follow-up children were not breastfed. The median DDS (IQR) was 3.0 (2.0, 4.0) (Table 2).

### Internal consistency of the components of ICFI for the cross-sectional study

The ICFI was positively associated with dietary diversity, food group frequency and feeding frequency and bottle feeding (*P* < 0.001) in the repeated cross-sectional study but not with breastfeeding as all were breastfed. However, the overall internal consistency estimated by the Cronbach-α coefficient was low (α = 0.45 in HS and α = 0.47 in PHS), but slightly better in the age group of 6–8 months in PHS (α = 0.58).

### Association between feeding practices and child nutrition

#### Repeated cross-sectional study

The ICFI and DDS were not associated with LAZ and WHZ among 6–12 month old children in both HS and PHS. After each component of the ICFI was assessed separately for its association with LAZ and WHZ, only bottle-feeding was associated with LAZ in PHS (β = −0.74, 95% CI: −1.20, −0.28; *P* = 0.002) in favor of those who were bottle fed (Table 3). Children with a DDS ≤ 2 account for 68 and 63% of all children in HS and PHS respectively. The odds of being stunted for children with a DDS ≤ 2 was 2.3 (95% CI: 1.1, 4.8; *P* = 0.03) times higher compared to their peers with DDS > 2 during the HS.

**Table 3:** Associations of ICFI and its components with LAZ and WLZ of children in the repeated cross-sectional study^b^

Variables	Harvest season (*n* = 296)					Preharvest season (*n* = 291)				
	*n*	Mean LAZ (SD)	*P*-value^*^	Mean WLZ (SD)	*P*-value^*^	*n*	Mean LAZ (SD)	*P*-value^*^	Mean WLZ (SD)	*P*-value^*^
Tertile of ICFI										
Low (reference)	152	−0.86 (1.41)		−0.66 (1.26)		132	−1.04 (1.23)		−0.78 (1.42)	
Medium	97	−0.66 (1.43)	0.63	−0.59 (1.30)	0.39	119	−1.20 (1.48)	0.41	−0.70 (1.36)	0.21
High	47	−0.74 (1.35)	0.78	−0.63 (1.26)	0.50	40	−0.60 (1.25)	0.40	−0.80 (0.99)	0.96
Dietary diversity (24 h)^a^										
Low (reference)	140	−0.77 (1.47)		−0.65 (1.25)		157	−1.13 (1.28)		−0.75 (1.34)	
Medium	99	−0.81 (1.38)	0.17	−0.62 (1.37)	0.50	75	−1.18 (1.51)	0.72	−0.87 (1.36)	0.74
High	57	−0.66 (1.31)	0.60	−0.63 (1.13)	0.42	59	−0.64 (1.18)	0.27	−0.61 (1.33)	0.43
Food group frequency (7d)^a^										
Low (reference)	62	−0.95 (1.51)		−0.67 (1.11)		34	−1.22 (1.31)		−0.79 (1.55)	
Medium	75	−0.74 (1.52)	0.46	−0.84 (1.39)	0.66	52	−1.09 (1.41)	0.78	−0.74 (1.30)	0.93
High	159	−0.72 (1.31)	0.28	−0.52 (1.26)	0.52	205	−1.01 (1.31)	0.24	−0.74 (1.32)	0.96
Bottle feeding^a^										
Yes (reference)	56	−0.75 (1.43)		−0.68 (1.29)		32	−0.21 (1.22)		−0.25 (1.15)	
No	240	−0.78 (1.41)	0.54	−0.62 (1.26)	0.39	259	−1.15 (1.31)	0.002	−0.80 (1.35)	0.15
Feeding frequency^a^										
Low (reference)	147	−0.88 (1.40)		−0.67 (1.30)		158	−1.09 (1.21)		−0.85 (1.41)	
Medium	63	−0.55 (1.24)	0.18	−0.36 (1.19)	0.05	54	−1.12 (1.47)	0.44	−0.48 (1.51)	0.07
High	86	−0.76 (1.53)	0.92	−0.78 (1.25)	0.87	79	−0.91 (1.50)	0.79	−0.74 (1.03)	0.61

*ICFI* Infant and child feeding index, *LAZ* length-for-age z-score, *WLZ* weight-for-length z-score

^*^ *P*-values are from multiple linear regression models controlling for confounding variables using backward stepwise selection. The association of individual components and LAZ and WLZ were modeled separately

^a^The low, medium and high categories are based on the scoring given for the each component of infant and child feeding index as 0, +1 and +2 respectively as described in Table 1

^b^All analyses are adjusted for child and maternal characteristics and household socioeconomic status in the multivariate regression model

In view of the absence of an important seasonal impact on the association between DDS, IFCI and child nutrition, we pooled the data of 6–12 month old children from the two seasons to increase power when testing the association of DDS and ICFI with nutritional status of children. We observed that the odds of being stunted were 1.70 (95% CI: 1.04, 2.71; *P* = 0.04) times higher among children with a DDS ≤ 2 compared to those with a DDS > 2. Moreover, being 9–12 months old [OR = 2.0; 95% CI: 1.24, 3.18; *P* < 0.01], having diarrhea [OR = 1.70; 95% CI: 1.05, 2.78; *P* = 0.04] and the presence of ≥2 under five children in the same household [OR =2.3; 95% CI: 1.24, 4.46; *P* = 0.01] were significantly associated with stunting (Table 4).

**Table 4:** Predictors of stunting in the pooled sample of all 6–12 months old children participating in the repeated cross-sectional study^a^

Variables	*n* ^c^ (%)	Odds Ratio	95% CI	*P*-value
DDS				
DDS > 2	204 (34.8)	Reference		
DDS ≤ 2	383 (65.2)	1.7	1.04, 2.71	0.04
Season				
HS	296 (50.4)	Reference		
PHS	291 (49.6)	1.0	0.64, 1.59	0.91
Human waste disposal				
Improper disposal	214 (36.5)	Reference		
Proper disposal	373 (63.5)	0.7	0.42, 1.01	0.05
Maternal schooling				
No formal schooling	489 (83.3)	Reference		
Attended formal school	98 (16.7)	0.5	0.25, 1.07	0.07
Age of the child				
6–8 months	223 (38.0)	Reference		
9–12 months	364 (62.0)	2.0	1.24, 3.18	˂0.01
Age of the mother				
< 20 years	92 (15.8)	Reference		
20–34 years	435 (74.7)	0.6	0.36, 0.98	0.03
≥ 35 years	55 (9.5)	0.7	0.27, 1.58	0.35
Diarrhea^b^				
No diarrhea	466 (79.7)	Reference		
Diarrhea present	119 (20.3)	1.7	1.05, 2.78	0.04
Under-five children in the HH				
0–1	145 (24.7)	Reference		
≥ 2	442 (75.3)	2.3	1.24, 4.46	0.01

*DDS* dietary diversity score, *HS* harvest season, *PHS* pre-harvest season, *HH* household

^a^Results from multiple binary logistic regression models using a backward stepwise selection of predictors

^b^Diarrhea in the previous 2 weeks of the studies

^c^Total numbers included in the analysis. Due to missing observations, the numbers for predicator variables “Age of the mother” and “Diarrhea” do not add up to 587

#### Follow-up study

The DDS of the 6–12 months old children during HS was positively associated with LAZ at follow-up period during PHS (β = 0.16; 95% CI: 0.01, 0.30; *P* = 0.03) (Table 5). The occurrence of diarrhea in the same season was negatively associated with LAZ (β = −0.35; 95% CI: −0.67, −0.03; *P* = 0.03). However, the DDS was not associated with LAZ and WHZ within seasons for the follow-up children.

**Table 5:** Multiple regression assessing the association between DDS and Length-for-Age Z-score adjusting for relevant confounding factors among the follow-up children in PHS^a^

Variables	*n* ^c^ (%)	β	95% CI	*P*-value
DDS during HS	266	0.16	0.01, 0.30	0.03
HAZ during HS	266	0.33	0.23, 0.43	<0.001
Age of the mother				
< 20 years	54 (21.1)	Reference		
20–34 years	181 (68.3)	0.43	0.01, 0.80	0.02
≥ 35 years	27 (10.6)	0.66	0.04, 0.13	0.01
Diarrhea^b^				
No diarrhea	199 (74.8)	Reference		
Diarrhea Present	67 (25.2)	−0.35	−0.67, −0.03	0.03
Maternal schooling				
No formal schooling	217 (81.6)	Reference		
Attended formal school	49 (18.4)	0.33	−0.05, 0.71	0.05
Socio-economic status				
Low	71 (26.9)	Reference		
Medium	94 (35.6)	0.41	0.06, 0.77	0.04
High	99 (37.5)	0.25	−0.10, 0.60	0.24

*DDS* Dietary diversity score, *HAZ* height-for-age z-score, *HS* harvest season

^a^Analysis of covariance was performed to determine predictors of growth at follow-up

^b^Diarrhea in the previous 2 weeks of the follow up study

^c^Total numbers included in the analysis. Due to missing observations, the numbers for predicator variables “Age of the mother” and “Socioeconomic status” do not add up to 266

## Discussion

The dietary diversity score during the HS was found to be an independent predictor of the subsequent 6 months of linear growth. Stunting was found to be associated with low DDS (≤2) only during HS. Moreover, we did not find such an association for ICFI. Within seasons, we did not find cross-sectional associations between DDS and ICFI and mean LAZ, WHZ and WAZ.

We previously found that children who consumed ≤2 food groups were likely to have inadequate micronutrient intake [15]. In the present analysis, the odds of being stunted was significantly higher for those who consumed ≤2 food groups in the previous 24 h. This was observed both among children in the HS and in the pooled data. Previous analyses also showed that DDS of ≤2 was an indicator of a low nutrient dense diet [20, 29]. An evaluation of the association of DDS with child anthropometry was not performed taking a DDS of ≤2 as a cutoff in those previous analyses. In one study in Mali, children in urban households with lowest DDS had higher risk of stunting and underweight [30]. Ruel and Arimond used tertiles of dietary diversity and the association with HAZ was observed in many of the countries for which the analyses was performed using DHS data [8]. Furthermore, it was observed that significant proportions of children from the African countries included in the analysis were consuming ≤2 food groups. A Kenyan study found an association between dietary diversity, calculated from 3 days average consumption of unique food groups, and five anthropometric indices. However, contrary to the present study, the samples of these previous studies were heterogeneous in terms of age groups and breast feeding status, which might explain the inconsistent results of associations between DDS and growth [31]. Younger children are more dependent on breast milk and their complementary food lack diversity compared to older children [32].

We did not observe a significant association between ICFI and LAZ after adjusting for confounders. This was also reported previously by studies conducted in rural West Africa [33] and China [11]. This finding, however, is in contrast to many other studies which showed significant associations of ICFI with LAZ [10, 12–14, 34]. Prior to the adoption of the WHO IYCF measurement indicators, the grouping of food items was done differently apart from differences in the number of food groups used to construct the DDS [35, 36], a component of ICFI. The numbers of components used to construct ICFI were also not uniform over different studies. For instance, we used five components whereas Sawadogo et al. [14], Ntab et al. [33] and Zhang et al. [11] used six, seven and eight components to construct the feeding index, respectively. Ntab et al. used an age group of 12–42 months for the analysis of the association between the ICFI and LAZ [33]. Moreover, dietary diversity is an important component in determining the relationship with child anthropometry when summary feeding measures are used [10, 14, 21]. The majority of the children (67%) in the present study consumed few food groups which in turn can affect ICFI’s discriminatory power and hence its association with LAZ [37]. All children in this study were breastfed and were given a high score for the breast feeding component in the construction of the ICFI. The latter scenario might have diluted the association of the ICFI with LAZ in comparison to a setting with a mixture of breastfed and non-breastfed children. Moreover, other studies have shown that breast feeding continues for an extended period of time in sick or malnourished children [38–41] who in turn were given a high score for the summary index.

There was no significant association between DDS and ICFI during the HS and PHS in 6–12 months old children participating in the repeated cross-sectional study. However, we found that DDS in HS was an independent predictor of linear growth during the 6 months of follow-up. This has also been observed by other researchers using a longitudinal ICFI (a summary indicator from repeated ICFIs over time) rather than an ICFI measured at the time of child anthropometry assessment. Such a composite ICFI was found to be associated with LAZ in children who were followed up for 7 months [21]. Another study in urban China showed that a longitudinal ICFI, but not dietary diversity, was associated with LAZ measured 1 year later [34]. This might indicate that of the positive evolution of feeding practices which led to positive changes in child anthropometry in the long term. DDS, rather than ICFI, was a predictor of growth for the follow-up children in the present study, but it is known that dietary diversity is an important factor in defining the relationship of ICFI with child anthropometry. Nevertheless, optimal growth is more of a cumulative effect of feeding practices involving several nutrients on top of other factors such as household food security, environmental hygiene and healthcare [3, 6, 10, 31, 36, 42].

Overall, child feeding practices in the present study are suboptimal compared to international recommendations with 88 and 67% of study children being introduced to complementary foods before the recommended age of 6 months during the HS and PHS respectively. Bottle feeding was relatively common and associated with LAZ in favor of bottle fed. On the contrary, premature introduction of complementary foods negatively affected linear growth in a longitudinal study among Vietnamese children [43]. According to an Ethiopian national survey, non-breastfed children were more likely to be fed with solid and semi-solid foods compared with breastfed children [4] but its association with growth was not elucidated. Breastfeeding for an extended period time was proposed to be associated with low HAZ in rural Senegal after mothers delayed weaning for stunted children [40, 41]. A stronger association was also found between dietary diversity and HAZ in non-breastfed children compared to breastfed peers [8, 38]. However, we were not able to demonstrate such associations as all of our children were breastfed.

This study has a number of strengths, but also several limitations that need to be addressed. First, the study was conducted in two distinct seasons in which intake of nutrients and LAZ scores significantly differed, which adds to the external validity of the overall analysis. Secondly, we followed children from the HS to another season to assess clustering of child feeding practices and its effect on growth in the long term. However, the internal validity of the components of the ICFI was found to be lower than what has been arbitrarily accepted. It is also impossible to rule out the day-to-day variability, random or reporting error in the assessment of child feeding practices which can result in misclassification and a reduced precision to detect an association [6]. Over-reporting of desirable feeding behaviors is a common occurrence in the measurement of feeding practices using recall techniques [6]. As it has been demonstrated previously [21, 34], it could have been more appropriate to construct the ICFI from feeding practice data collected from the same subjects at different time points to evaluate the occurrence of clustering of feeding behaviors and their impact on growth or nutritional status in the long term.

## Conclusions

In conclusion, DDS and ICFI are not unequivocally related to child growth. Whereas DDS during the HS was able to predict longer term growth and only very poor dietary diversity was found to be associated with child stunting during HS and in the pooled data. Our data suggests the use of a minimal DDS (≤2) rather than a mean DDS as an indicator for suboptimal child growth. We did not find any association between ICFI and child growth, which might suggest that there is a room to add other dimensions of appropriate practices of complementary feeding, such as responsiveness and hygienic preparation and storage of complementary foods.

## Additional file

Additional file 1: Survey questionnaire. Questionnaire administered to the study participants to collect data on feeding patterns and practices and child growth. (DOC 130 kb)

## Abbreviations

DDS: Dietary diversity score
EDHS: Ethiopia Demographic and Health Survey
HAZ: Height-for-age z-score
HH: Household
HS: Harvest Season
ICFI: Infant and child feeding index
IQR: Interquartile range
LAZ: Length-for-age z-score
MMDA: Mean micronutrient density adequacy
OR: Odds ratio
PHS: Pre-harvest season
SD: Standard deviation
SES: Socioeconomic status
WAZ: Weight-for-age z-score
WHO: World Health Organization
WHZ: Weight-for-height z-score
WLZ: Weight-for-length z-score
