# Supplementary material for: Feeding practices and growth among young children during two seasons in rural Ethiopia
Source: BMC Nutr. 2017 Apr 24;3:39. doi: 10.1186/s40795-017-0158-y (PMC7050872; doi:10.1186/s40795-017-0158-y)
Supplement: Supplementary file 1 — Survey questionnaire. Questionnaire administered to the study participants to collect data on feeding patterns and practices and child growth. (DOC 130 kb) [file 40795_2017_158_MOESM1_ESM.doc]

**Survey questionnaire**

**Questionnaire administered to the study participants to collect data on feeding patterns and practices and child growth**

*Dear interviewer: If verbal consent is granted, begin the interview. If the respondent does not agree to continue, thank her and go to the next interview. Discuss this result with your supervisor for a future revisit.*

| Result of first visit | 1. Completed 2. Not willing | 1. Was not available 2. Interrupted | Date of appointment for revisit ……………………. |
| --- | --- | --- | --- |
| Result of second visit | 1. Completed 2. Not willing | 1. Was not available 2. Interrupted | Date of appointment for revisit ……………………. |
| Result of third visit | 1. Completed 2. Not willing | 1. Was not available 2. Interrupted |  |

| 1. Background Information | | |
| --- | --- | --- |
|  | Child ID | |__|__|| __| |
|  | Name of the Woreda |  |
|  | Name of the Kebele |  |
|  | Name of Gere |  |
|  | Name of the “got” |  |
|  | House number |  |
|  | Name of the household head |  |

| 1. Socio economic and demographic characteristics of caregivers | | | |
| --- | --- | --- | --- |
|  | Questions | Coding categories | skip |
|  | Relation of the respondent to the index infant | Mother . 1  Elder sibling 2  Care giver 3  Other 4 | . |
|  | What is the age of the mother/care giver ( in years) | ____________ |  |
|  | Marital status of the mother/ care taker | Married 1  Single 2  Divorced 3  Separated 4  Widowed 5 |  |
|  | Religion of the mother/care giver | Islam 1  Orthodox 2  Protestant 3  Catholic 4  Other ( specify)__________ |  |
|  | Ethnicity of the mother or care taker | Oromo 1  Amhara 2  Tigre 3  Answer refused 77  Other ( specify)__________ |  |
|  | Educational status of the mother /care taker | Unable to read and write 1  Only read and write 2  Attended formal education  (specify the level of education attained) _____________ |  |
|  | Educational status of the father | Unable to read and write 1  Only read and write 2  Attended formal education  ( Specify the level of education attained) ____________________ |  |
|  | How many children under 5 years of age usually live in this household? | Number children under 5 years |  |
|  | What is the current main source of drinking water for members of your household?  *Circle ONLY ONE answer* | Piped water 1  Tube well or borehole 2  Protected well 3  Unprotected well 4  Water from protected spring 5  Water from unprotected spring 6  Rainwater 10  Surface source (stream, river,  pond, lake, canal) 12  Don’t know 98  Answer refused 77  Other (specify) 96  _______________________________ |  |
|  | Do you treat your water in any way to make it safer to drink?  *Circle ONLY ONE answer*  *If yes, continue.*  *If other answer, jump to question 30.* | Yes 1  No 2  Don’t know 98  Answer refused 77 |  |
|  | What do you do now to the water to make it safer to drink?  *Circle ALL applicable answers* | Boil 1  Add bleach, chlorine or Agar 2  Mix with leaves 3  Strain it through a cloth 4  Use a water filter 5  Let it stand and settle 6  Answer refused 77  Don’t know 98  Other (specify) 9  _______________________________ |  |
|  | Where do members of your household usually go to relieve themselves?  *Circle ONLY ONE answer* | Flush toilet 1  Pit latrine with slab …………………2  Pit latrine without slab/open pit…….3  Composting toilet 4  Bush or field 5  On ground within compound 6  Answer refused 77  Other (specify) 96  _______________________________  Don’t know 98 |  |
|  | What is the material from which your house is made up of? | Materials |  |
|  | - 1. Roof   *Record observation* | Thached/Grass 1  Corrugated iron sheet 2  Other ( specify) 96 |  |
|  | - 1. Wall   *Record observation* | Cement and wood 1  Cement, wood and mud 2  Wood and mud 3  Stone and mud 4  Wood 5  Other ( specify) 96 |  |
|  | - 1. Floor   *Record observation* | Cemented 1  Ceramic tiles 2  Carpet 3  Dung 4  Wooden 5  Mud 6 |  |
|  | What is now the primary source of income for this household?  *Circle ONLY ONE answer* | Farming, including cash crops 1  Livestock 2  Employment/salary 3  Petty trading (including sale of fire-  wood, charcoal, grass, local brewery)  Daily labor 5  Handicrafts/artisan 6  Remittances 7  Answer refused 77  Don’t know 98  Other (specify) 96  ______________________________ |  |

|  | I will now mention some animals, and I would like you to tell me how many animals of each type you have  *Fill in NUMBER of each type of animal* | Plow oxen:  Cows:  Hephers:  Calves:  Bulls:  Sheep and goats:  Horses, donkeys, mules:  Chickens: |  |
| --- | --- | --- | --- |
|  | How much land does your household own?  Write in number of local units and the name of the local unit | Number of local units:  Name of local unit:  “Fechasa”………... 1  “Timad” ………… 2  “Hectare”………... 3  Other ………….. ..........96  Specify: ________________  Don't know how much land………...98 |  |
|  | Does your household have:  *Circle 1 Or 2 For Each Item* | Yes No  Electricity/generator: 1 0  A sewing machine: 1 0  A radio: 1 0  A television: …………........................ 1 0  A mobile telephone: 1 0  A fixed telephone: ……………………1 0  A refrigerator: 1 0  A table: ………………………………...1 0  A chair:…………………………………1 0  A bed: ………………………………….1 0  An electric “mitad”: 1 0  A bicycle:………………………………1 0  A motorcycle: ………………………….1 0  A “Bajaj”: ……………………………...1 0  A kerosene or pressure lamp: …………1 0  A kerosene /electric stove:……………..1 0 |  |

| 1. Child characteristics and child feeding practice of the caregiver | | | | |
| --- | --- | --- | --- | --- |
|  | Ethiopian date of birth?  Reported by the mother  Recorded from birth certificate  Recorded from immunization card  Estimated using local events calendar | 1. |__|__|/|__|__|/|__|__| (dd/mm/yy) 2. |__|__|/|__|__|/|__|__| (dd/mm/yy) 3. |__|__|/|__|__|/|__|__| (dd/mm/yy) 4. |__|__|/|__|__|/|__|__| (dd/mm/yy) |  | |
|  | Sex of the child | Male…....1  Female... 0 |  | |
|  | Is the child ever breastfed? | Yes…….. 1  No………0 | 0⇨ 308 | |
|  | Is the child currently breastfed? | Yes…….. 1  No ……...2 |  | |
|  | Was the child fed only breast milk over the last 24 hours? | Yes ……..1  No………2 |  | |
|  | How many times did you breastfed the child over the last 24 hours (day and night)?  *Probe the mother: How many times did you breastfeed last night between sunset and sunrise and?*  *How many times did you breastfeed yesterday during the daylight hours?* | |__|__|Times |  | |
|  | For how long after birth was the child fed only breast milk?  *( Probe the mother: for the additional foods, water, tea, fenugreek , etc,)*  *Please use the local calendar to assist the mother to remember the duration* | |__|__|-days/ months  Don’t know--------------- |  | |
|  | Have you given additional food other than breast milk over the past seven days including yesterday? | Yes...... 1  No....... 0 |  | |
|  | Now I would like to ask you about the types of foods [name] has been fed over the last seven days, including yesterday. How many days during last seven days was [*name*] given each of the following? FOR EACH ITEM GIVEN AT LEAST ONCE IN LAST SEVEN DAYS, ASK: In total, how many times yesterday during the day or at night was [name] given: | Last 7 days number of days | Yesterday/ last night number of times the item was given | |
|  | **Grains /tubers/ roots;**   1. Any porridge or gruel (made from grains other than teff)? 2. Bread, pasta, rice, biscuits, cookies or any other food made from oats, maize, barley, wheat, sorghum, millet, or other grain? 3. Any food made from teff, like injera, kita or porridge? 4. Any white potatoes, white yams, bulla, kocho, cassava, or any other foods made from roots?   **Legumes/nuts;**   1. Any foods made from beans, peas, chickpea, lentils or pulses? 2. Any nuts or seeds such as peanuts (*lewiz*), sesame (s*elit*) or sunflower seeds?   **fruits and vegetables**   1. Any pumpkin, carrots, squash or sweet potatoes that are yellow or orange inside? 2. Any ripe mangoes, papayas? 3. Any dark green, leafy vegetables like kale (gomen), spinach (kosta), lettuce ( salata)   **Other fruits and vegetables**   1. Any other fruits or vegetables?   **Flesh foods;**   1. Any liver, kidneys, heart or other organ meats? 2. Any beef, lamb, goat? 3. Any chicken, duck or other birds? 4. Any fresh or dried fish or sea foods   **Eggs**   1. Any eggs?   **Milk and dairy products**   1. Any cheese or yogurt? 2. Any Cerifam, Fafa, Milupa, Babylac, Mother's Choice or other commercially fortified baby food?   **Fats and oils**   1. Any food made with oil, fat, butter, or galighee, red palm oil. | 1. |__|__| DK ……8 2. |__|__| DK ……8 3. |__|__| DK ……8 4. |__|__| DK …… 8 5. |__|__| DK …… 8 6. |__|__| DK …… 8 7. |__|__| DK …… 8 8. |__|__| DK …… 8 9. |__|__| DK …… 8 10. |__|__| DK …… 8 11. |__|__| DK ……8 12. |__|__| DK …….8 13. |__|__| DK …… 8 14. |__|__| DK ……. 8 15. |__|__| DK …….8 16. |__|__| DK …….8 17. |__|__| DK …….8 18. |__|__| DK …….8 | 1. |__|__| 2. |__|__| 3. |__|__| 4. |__|__|      1. |__|__| 2. |__|__|      1. |__|__| 2. |__|__| 3. |__|__| 4. |__|__|      1. |__|__| 2. |__|__| 3. |__|__| 4. |__|__| 5. |__|__|      1. |__|__| 2. |__|__| 3. |__|__| | |
|  | How many times was [name] fed mashed or pureed food or solid or semi-solid food yesterday during the day or at night? (If 7 or more times, record ‘7’) | No. of times |__|__|  DK ………… 98 | |  |
|  | Do you make special processing to improve the nutritional value / taste or other qualities of the additional food you give to your child? | Yes ………………..1  No …………………0 | | 0⇨ **314** |
|  | What processing techniques you use at home to prepare food for your child? | Removal of bran of grains and cereals………………………………..1  Fermentation…………………………2  Soaking ………………………………3  De-hulling ……………………………4  Others………………………………...96  Specify_________________________  Answer refused……………………….77 | |  |
|  | Did (*name*) drink anything from a bottle with a nipple yesterday or last night? | Yes ……………………………….1  No ………………………………..0 | |  |

| 1. Child morbidity | | |
| --- | --- | --- |
|  | Try to remember the last two weeks. During this period, has *(name)* had diarrhea?  **Definition**:  *diarrhea is defined as having three or more loose stools during a 24-hour period*  *circle only one answer* | Yes 1  No 2  Answer refused 7  Don´t know 8 |
|  | Try to remember the last two weeks again. Has *(name)* had a cough?  *circle only one answer* | Yes 1  No 2  Answer refused 7  Don´t know 8 |
|  | Try to remember the last two weeks again. Has *(name)* had difficulty breathing?  *Difficulty of breathing involves : chest in-drawing, grunting, and unusually rapid breathing*  *circle only one answer* | Yes 1  No 2  Answer refused 7  Don´t know 8 |
|  | Try to remember the last two weeks again; has *(name*) had a fever?  *circle only one answer* | Yes 1  No 2  Answer refused 7  Don´t know 8 |

| 1. Anthropometric measurement of the child | | | |
| --- | --- | --- | --- |
|  | Child ID | |__|__|| __| |  |
|  | Child’s weight. | Kilograms (kg) |__|__|.|__| |  |
|  | Child’s length  *Please measure length in recumbent position* | Length (cm) |__|__|| __|.|__| |  |
|  | Oedema | Present …………………1  Absent…………………..2  Not checked……………..3 |  |

| Name and signature the interviewer: |  |
| --- | --- |
| Name and signature of the supervisor |  |
| Day/month/year of interview: | |__|__|/|__|__|/|__|__| (dd/mm/yy) |

***Dear interveiwer: Thank the mother/caregiver and fill in the appropriate box about the status of the interview***
